# P2X7 receptor isoform B is a key drug resistance mediator for neuroblastoma

**DOI:** 10.3389/fonc.2022.966404

**Published:** 2022-08-25

**Authors:** Vanessa Fernandes Arnaud-Sampaio, Carolina Adriane Bento, Talita Glaser, Elena Adinolfi, Henning Ulrich, Claudiana Lameu

**Affiliations:** ^1^ Biochemistry Department, Institute of Chemistry, University of Sao Paulo, Sao Paulo, SP, Brazil; ^2^ Section of Experimental Medicine, Department of Medical Sciences, University of Ferrara, Ferrara, Italy

**Keywords:** P2X7 receptor isoforms, chemoresistance, childhood cancer, cancer, drug efflux, cancer stem cells, autophagy, epithelial-mesenchymal transition

## Abstract

Drug resistance is a major challenge for all oncological treatments that involve the use of cytotoxic agents. Recent therapeutic alternatives cannot circumvent the ability of cancer cells to adapt or alter the natural selection of resistant cells, so the problem persists. In neuroblastoma, recurrence can occur in up to 50% of high-risk patients. Therefore, the identification of novel therapeutic targets capable of modulating survival or death following classical antitumor interventions is crucial to address this problem. In this study, we investigated the role of the P2X7 receptor in chemoresistance. Here, we elucidated the contributions of P2X7 receptor A and B isoforms to neuroblastoma chemoresistance, demonstrating that the B isoform favors resistance through a combination of mechanisms involving drug efflux *via* MRP-type transporters, resistance to retinoids, retaining cells in a stem-like phenotype, suppression of autophagy, and EMT induction, while the A isoform has opposite and complementary roles.

## Introduction

Drug resistance is undoubtedly the greatest challenge in the fight against cancer (1). Although new treatment options are progressively developed, few surviving cancer cells are enough to promote disease relapse, and the multiple factors that influence the ability of cancer cells to evade therapy are highly complex. In the case of neuroblastoma (NB), a childhood tumor affecting cells of the sympathetic peripheral nervous system, nearly 50% of high-risk patients either relapse or do not respond to first-line therapy protocols (2). Therefore, the investigation of molecular targets involved in cancer cell fate determination, which shift cells toward either death or survival, is crucial for advancing cancer research.

The P2X7 receptor, an ion channel of the P2X family of ATP-gated ionotropic purinergic receptors, is of particular interest given its long-known cell death-inducing properties (3). Thus, it has been previously hypothesized that P2X7 receptor stimulation in cancer cells could present a survival challenge, contributing to successful cancer therapy. Indeed, P2X7 receptor upregulation in brain tumor cells is a good prognostic predictor for radiation therapy response, and P2X7 receptor agonists have been shown to potentialize the cytotoxic effect of anticancer agents (4–6).

The cell death-promoting role of the P2X7 receptor depends on the formation of a large nonselective membrane pore that is permeable to molecules of up to 900 Da. Macropore opening is triggered by sustained receptor stimulation and leads both to altered membrane permeability, allowing the influx of large molecules, and to ATP efflux (7), increasing the ATP concentration in the extracellular space.

However, very high concentrations of extracellular ATP (5-10 mM) are required to activate the cytotoxic activity of the P2X7 receptor, and although these concentrations are probably achieved following classical anticancer therapies that lead to necrosis, such as chemotherapy or radiotherapy, they are rarely present in the untreated tumor microenvironment (TME) due to the activity of ubiquitous ectonucleotidases (3, 8). Therefore, the P2X7 receptor is usually active only as an ion channel within the TME, and as such, it has been shown to promote cell growth, neovascularization, matrix degradation and metastasis in various solid and liquid tumor models, including neuroblastoma (3, 9–11).

In addition to the biphasic gating of the P2X7 receptor ion channel, the existence of distinct splice variants of the *p2rx7* gene also plays a role in determining distinct cellular responses (3). Among the isoforms generated by alternative splicing of the human *p2rx7* gene, the A and B isoforms are the only well-characterized functional ion channels and are ubiquitously expressed (12). While P2X7A corresponds to the full-length variant, P2X7B arises from the retention of an intron containing a stop codon, which shortens the protein length (13, 14). This truncated version lacks the C-terminal tail that has been described as crucial to macropore opening. However, the P2X7B variant is still functional, as it retains the capability of opening the ion channel, leading to downstream cellular effects (14, 15). In previous studies, the P2X7A variant has produced death-promoting antitumor effects, while the P2X7B variant has been implicated in the trophic, metastatic and resistance-prone properties of tumor cells (16–18). Furthermore, our group has observed that the malignancy of NB cells is related to increased P2X7B expression (19).

Therefore, we aimed to resolve the differential contributions of P2X7 receptor splice variants to the drug resistance of NB by further analyzing the mechanisms involved. Our results point to multifaceted drug resistance, comprising many mechanisms that together build up a consistent resistant phenotype. On the one hand, we demonstrated that P2X7A favors retinoid sensitivity to NB-cell differentiation and autophagy and downregulates efflux pumps. On the other hand, we showed the complementary role of the P2X7B isotype in suppressing autophagy, inducing drug efflux, and favoring EMT. The obtained results are highly relevant for explaining the dual role of the P2X7 receptor in tumor biology, paving the way for innovative therapies for chemoresistant tumors.

## Materials and methods

### 1. Cell lines

ACN human neuroblastoma cells derived from bone marrow metastasis (RRID : CVCL_1068) and HEK 293 cells (ATCC^®^ CRL-1573™) were genetically modified as previously described (16, 20). ACN cells were silenced with shRNAs from OriGene™ as detailed in **Table 1**, resulting in three derived cell lines.

**Table 1:** Target sequences of shRNAs used for ACN cell silencing.

	Target sequence (5’-3’)	Predicted phenotype
**shRNA1** **c.n. TI202483**	ACGTTTGCTTTGCTCTGGTGAGTGACAAG	P2X7A^-^/B^-^
**shRNA2** **c.n. TI202486**	CATTAGGATGGTGAACCAGCAGCTACTAG	P2X7A^-^/B^+^
***scrambled s*hRNA** **c.n. TR30012**	–	P2X7A^+^/B^+^

HEK 293 cells, which do not natively express P2X receptors, were transfected for P2X7 receptor A or B isoform expression, resulting in the following derived cell lines: HEK 293-A, expressing P2X7A; HEK 293-B, expressing P2X7B; and HEK 293-*mock*, not expressing any P2X7 receptor isoform. Procedures for obtaining transfection constructs are described in detail elsewhere (16).

### 2. Cell culture

The culture medium for ACN cells consisted of RPMI 1460 medium supplemented with 10% bovine fetal serum, 1% NEAA 100X solution (Gibco™, c.n. 15140122), 100 U/mL penicillin, 100 mg/mL streptomycin, and the clone selection antibiotic puromycin at a 500 nM final concentration. Cells were kept at 37°C and 5% CO_2_.

HEK 293 cells were cultivated in DMEM/F12 culture medium supplemented with 10% bovine fetal serum, 100 U/mL penicillin, 100 mg/mL streptomycin, and specific clone selection antibiotics (G48 sulfate at 200 ng/ml for HEK 293-A and HEK 293-*mock* cells and hygromycin B at 100 µg/ml for HEK 293-B cells). Cells were kept at 37°C and 5% CO_2_.

### 3. Cell line characterization

For cell line validation and characterization of P2X7 receptor isoform expression patterns, PCRs were performed.

For ACN cells, TaqMan™ real-time RT–PCR assays were conducted. After RNA extraction and cDNA synthesis, as described below in the specific section, Taqman™ reactions were prepared containing 1 µl of cDNA, 1X Taqman™ Fast Advanced Master Mix, Applied Biosystems™, 1.8 µM final concentration of primers, 0.5 µM of FAM-MGB probe, and 1X GAPDH predesigned Taqman™ assay Hs02758991_g1 VIC-MGB. Cycling conditions consisted of 2 min at 50°C, 2 min at 95°C, followed by 40 cycles of 1 s at 95°C plus 20 s at 60°C in the thermocycler StepOnePlus^®^, Life Technologies. For quantification, we used the calculation method 2^-ΔΔCt^, normalizing *scrambled*-transfected sample values to 1.

The probe sequences were as follows: P2X7A 5′ CACAGCGGCCAGACCG 3′ - 6FAM; P2X7B 5′ ACAAGCGCTGCGTTAGT 3′ - 6FAM.

The primers used were as follows: P2X7A Forward 5′ CGGCTCAACCCTCTCCTACT‐3′; P2X7A Reverse 5′ GGAGTAAGTGTCGATGAGGAAGTC-3′; P2X7B Forward 5′GGAAAATGGTTTGGAGAAGGAAGTG-3′; and P2X7B Reverse 5′CGATGAGGAAGTCGATGAACACA-3′.

For HEK 293 cells, conventional reverse transcription PCRs were used. After RNA extraction and cDNA synthesis, as described below in the specific section, PCRs were prepared with 1.25 U Taq DNA polymerase, Invitrogen™ in 1X Buffer, 2 mM MgCl_2_, 0.2 mM dNTPs, forward and reverse primers at 0.5 µM each, and 1 µL of cDNA in a 25 µL reaction. After denaturation at 94°C for 2 min, the reaction proceeded with 40 cycles of 94°C for 1 min + 50°C for 1 min + 68°C for 2 min. Finally, a cycle of 68°C for 10 min was conducted to guarantee the final extension of DNA strands.

The obtained products were subjected to electrophoresis in a 1.5% agarose gel prepared with TAE buffer. Samples were run at 80 V for 30 min, and images were obtained on a UV transilluminator.

The primers used were as follows: P2X7B Forward: 5’CCCATCGAGGCAGTGGA 3’; P2X7B Reverse: 5’ TAAAGCATGGAAAAGAGAATCTC 3’; panP2X7 Forward: 5’ AGATCGTGGAGAATGGAGTG 3’; panP2X7 Reverse: 5’ TTCTCGTGGTGTAGTTGTGG 3’; GAPDH Forward: 5’ CCTCTGACTTCAACAGCGAC 3’; GAPDH Reverse: 5’ CATGACAAGGTGCGGCTCCC 3’.

### 4. Tumorsphere culture

For culturing cells as tumorspheres, cells were seeded in 0.2% F-127 pluronic acid-coated hydrophobic suspension dishes in defined DMEM High medium containing 20 ng/ml EGF, 20 ng/ml FGF and 1X N-2 supplement, Gibco™, for at least 96 h. For flow cytometry, tumorspheres were dissociated with 2 mM EDTA and vigorously pipetted.

### 5. Cell viability – alamarBlue^®^

For cell viability assessments, cells were seeded on 96-well plates with black borders and clear bottoms at a density of 10^4^ cells per well with complete medium overnight. After adherence and 2 h of starvation in serum-depleted medium, the chemotherapy drugs vincristine (300 nM) or doxorubicin (100 µM) were added, combined or not with treatments (described in **Table 2** and detailed in each figure), in supplemented medium without clone selection antibiotics (unless specifically stated) for 48 h.

**Table 2:** Details of pharmacologic treatments other than chemotherapy drugs.

	Description	Concentration
**ATP**	P2X7 receptor agonist	1 mM
**BzATP**	P2X7 receptor agonist	100 µM
**γ-S-ATP**	P2X7 receptor agonist	100 µM
**A438079**	P2X7 receptor antagonist	10 µM
**BBG**	P2X7 receptor antagonist	300 nM
**Retinoic Acid**	Neural differentiation inducer	5 µM
**TGF-β**	EMT inducer	5 ng/ml
**EGF**	EMT inducer	50 ng/ml
**Probenecid**	MRP-type inhibitor	1 mM
**Verapamil**	Pgp inhibitor	5 µM
**Ko143 hydrate**	BCRP inhibitor	30 nM
**Rapamycin**	Autophagy inducer	200 nM

For comparison between culture conditions, cells were also subjected to 2 h of starvation in serum-depleted medium and then incubated with the tested culture medium. To observe the effects of nutrient availability on drug resistance, a culture condition gradient composed of four conditions was tested: EBSS buffer, EBSS buffer + 2 g/dL glucose, MEM-EBSS medium, and MEM-EBSS + 10% FBS. For autophagy induction through serum starvation, groups consisted of a control group cultured in RPMI 1640 10% FBS and a starved group cultured in RPMI 1640 without FBS.

After specific interventions, the cells were incubated for a 2 h period with a 1:10 alamarBlue^®^ solution prepared in culture medium protected from light at 37°C and 5% CO_2_. Fluorescence readings were performed in FlexStation III, Molecular Devices™, at 530-560/590 nm excitation/emission wavelengths, as specified by the manufacturer. Cell viability values were compared to the control group, considered as 100%.

When analyzing the influences of a specific drug (detailed in **Table 2**) or culture condition on the effects of vincristine or doxorubicin, calculations were performed as described. First, the cell viability of the group treated with the additional drug alone was calculated considering the cell viability of the untreated group of the same cell line as 100%. A one-tailed paired *t* test analysis determined whether the treatment alone was cytotoxic considering a confidence interval of 95%.

If *p*>0.05: Cell viability of all groups in the analysis was calculated considering the cell viability of the untreated group as 100% and compared altogether using one- or two-way ANOVA variance analysis, according to experimental design.

If *p*<0.05, additional treatment was considered cytotoxic, and as such, groups that received the cytotoxic additional drug were normalized considering the cell viability of the group treated exclusively with the cytotoxic drug as 100%, and related groups were compared for statistically relevant differences using *t* tests (when only two samples) or ANOVA, according to the experimental design.

### 6. Cell death – propidium iodide (PI)

For cell death analysis, cells were seeded in 12-well plates at a density of 3 x 10^5^ cells per well with complete medium and incubated overnight. After adherence and 2 h of starvation in serum-depleted medium, chemotherapy drugs were added, combined or not with the treatments detailed in **Table 2**, in supplemented medium without clone selection antibiotics (unless specifically stated) for 48 h. After detachment with 2 mM EDTA, cells were washed in PBS, passed through a 40 µm cell strainer, and stained for 10 min with a 2 µg/ml PI solution in PBS at 4°C. The positive control consisted of a mixed cell sample incubated in 2 µg/ml PI + 0.1% Triton X-100 PBS solution, while the negative control was a mixed cell sample incubated in pure PBS.

Cell death rates were determined in an Attune™ Acoustic Focusing Cytometer, Life Technologies™, with minimal acquisition of 50,000 events per sample at 200 µL/min flow speed. The obtained data were analyzed in FlowJo software (BD Biosciences).

### 7. Dose–response curves

To characterize the response pattern of the employed cell lines to the chemotherapy drugs, cell viability drug-response curves were performed employing crescent drug concentrations in intervals of 0.5 log units: 0-100 µM for vincristine, 0-10 mM for doxorubicin, and 0-30 mM for cyclophosphamide. For cell death (PI staining) drug-response curves, the concentration ranges were 0-10 µM for vincristine, 0-100 µM for doxorubicin, and 0-10 mM for cyclophosphamide. IC50 and EC50 concentrations were calculated based on nonlinear regression analysis performed in GraphPad Prism 5™ software.

### 8. RNA extraction

For relative gene expression analysis, cells were seeded on 6-well plates at a density of 3 x 10^6^ cells per well. After adherence and 2 h of starvation in serum-depleted medium, the respective treatments were applied in supplemented medium without clone selection antibiotics (unless specifically stated).

The cells were then washed with PBS, collected directly in TRIzol and frozen at -80°C until extraction with cold chloroform, precipitated in isopropanol and washed in 75% ethanol, as instructed by the manufacturer’s protocol.

### 9. cDNA synthesis and relative expression analysis – RT–qPCR

Reverse transcription reactions using both oligoDT and random hexamers were performed after treating RNA samples with DNAse. We used a RevertAid^®^ reverse Transcription Kit from Invitrogen (Thermo Fisher) to synthesize cDNA from 2 µg of purified RNA from each sample following the manufacturer’s protocol. Cycling conditions consisted of 5 min at 65°C, followed by 10 min at 25°C, 42°C for 60 min and 70°C for 10 min.

Primer pairs with the sequences specified in **Table 3** and SYBR Green Master Mix 2X reagent were incubated with 1 µl of 10X diluted cDNA synthesis product. The cycling conditions for amplification were 95°C for 1 min for denaturation, 40X 95°C for 30 s, and 60°C for 1 min, followed by melting curve analysis performed in 3°C increments. The thermocycler StepOnePlus^®^, Life Technologies, was used. For quantification, we used the calculation method 2^-ΔΔCt^, normalizing control sample values to 1. As an endogenous control, primers targeting the EMC7 gene were employed.

**Table 3:** qPCR primer sequences.

Target	Sequence (5’→ 3’)
**P-gp (ABCB1) Forward**	CGTGGGGCAAGTCAGTTCA
**P-gp(ABCB1) Reverse**	TCCTTCCAATGTGTTCGGCA
**MRP1 (ABCC1) Forward**	ACTAGGAAGCAGCCGGTGAA
**MRP1 (ABCC1) Reverse**	CTTCTGTGGGGACTTGACGA
**BCRP (ABCG2) Forward**	TGAAAAGGATGTCTAAGCAGGGA
**BCRP (ABCG2) Reverse**	GCAGGCCCGTGGAACATAA
**N-cadherin Forward**	GCCCAAGACAAAGAGACCCA
**N-cadherin Reverse**	TCAACTTCTGCTGACTCCTTCA
**Fibronectin Forward**	TGGGCAACTCTGTCAACGAA
**Fibronectin Reverse**	CCACTCATCTCCAACGGCAT
**Nanog Forward**	AGAAAGAGGTCTCGTATTTGCTG
**Nanog Reverse**	ACACTCGGTGAAATCAGGGT
**Twist2 Forward**	GACAGCAGTGACATCGGACA
**Twist2 Reverse**	GACCCAGAAGAAAAATCCAAACAGA
**Twist1 Forward**	CCACTGAAAGGAAAGGCATCAC
**Twist1 Reverse**	TATGGTTTTGCAGGCCAGTT

### 10. Protein expression analysis – flow cytometry

Protein expression levels were quantified by flow cytometry. Cells were detached from culture plates with 2 mM EDTA, fixed in PFA 4% for a minimum of 1 h, and blocked/permeabilized in 4% FBS + 0.1% Triton X-100 PBS solution. An overnight incubation was then performed with primary antibodies (Abcam^©^ ab129450, ab3380, ab32574, ab207612, and ab1316; Molecular Probes^®^ L10382) at a 1:100 dilution in 1% FBS PBS 1X solution, followed by staining with Alexa Fluor^®^ 555 anti-mouse or Cy5^®^ anti-rabbit secondary antibodies in 4% FBS + 0,1% Triton X-100 PBS 1X solution for 15 min.

Flow cytometry measurements were performed in an Attune™ Acoustic Focusing Cytometer, Life Technologies™, with BL-2 or RL-1 filter settings (Alexa Fluor^®^ 555 anti-mouse or Cy5^®^ anti-rabbit, respectively). Fluorescence thresholds were determined by negative samples incubated with secondary antibody in FlowJo software (BD Biosciences).

### 11. Cell cycle analysis

Cells were detached from culture plates with 2 mM EDTA, washed in PBS, fixed in 70% ethanol for 1 h at 4°C, passed through a 40 µm cell strainer, blocked and permeabilized in 1% FBS + 0.1% Triton X-100 PBS 1X solution for 30 min with agitation. Next, the cells were incubated with anti-ki67 Millipore^®^ antibody (AB9260) in 1% BFS + 0,1% Triton X-100 PBS 1X solution for 45 min with agitation and further incubated with Alexa Fluor^®^ 555 anti-rabbit IgG antibody for 15 min. Finally, the cells were washed and incubated with RNAse A 50 µg/ml solution in 0.1% Triton X-100 + 0.5% Tween 20 PBS 1X solution for 15 min. Populations were measured in an Attune™ Acoustic Focusing Cytometer, Life Technologies™, with minimal acquisition of 50,000 events per sample at 200 µL/min. The obtained data were analyzed using the FlowJo software (BD Biosciences).

### 12. Efflux activity – Hoechst 33342^®^ staining followed by image cytometry (TissueFAXS^®^)

For efflux activity assessments, cells were seeded on 96-well plates with black borders and clear bottoms at a density of 10^4^ cells per well with complete medium overnight.

Efflux activity by ATP-binding cassette (ABC) transporters was measured using the dye Hoechst 33342 at a 100 ng/ml solution prepared in culture medium, followed by incubation for 30 min and fixation in 4% PFA. A propidium iodide solution (PI) at 3 µg/mL was used to stain cell nuclei. Plates were scanned in the TissueFAXS^®^, TissueGnostics, fluorescence microscope, and cytometric quantifications were performed by StrataQuest^®^ software.

Detection of PI staining by the nuclei mask tool in StrataQuest^®^ software allowed mapping of the cell nuclei area in the images. Next, the fluorescence intensity for Hoechst 33342 was measured in the determined area and visualized as dot plots. As a negative control, samples stained with PI only were employed, while a positive control was prepared by cell fixation with 4% PFA before Hoechst 33342 staining. Thus, the method identified low Hoechst 33342-stained populations that were able to expel the dye.

### 13. Drug interaction analysis

For drug interaction analysis, we employed the Bliss Independence model followed by *t* tests comparing transformed values of predicted and observed survival fractions to determine the statistical relevance of the observations, as described elsewhere (21).

### 14. Statistical analysis

For the cell viability and efflux assays, variances among groups were compared by paired *t* tests and one- or two-way ANOVA according to the experimental design followed by Bonferroni post tests, considering a 95% confidence interval. For relative expression analysis, one-way ANOVA was employed, followed by Bonferroni post test, also considering the 95% confidence interval.

## Results

### The P2X7B isoform is implicated in drug resistance, while the P2X7A variant is related to cell death induction

**Figure 1:**
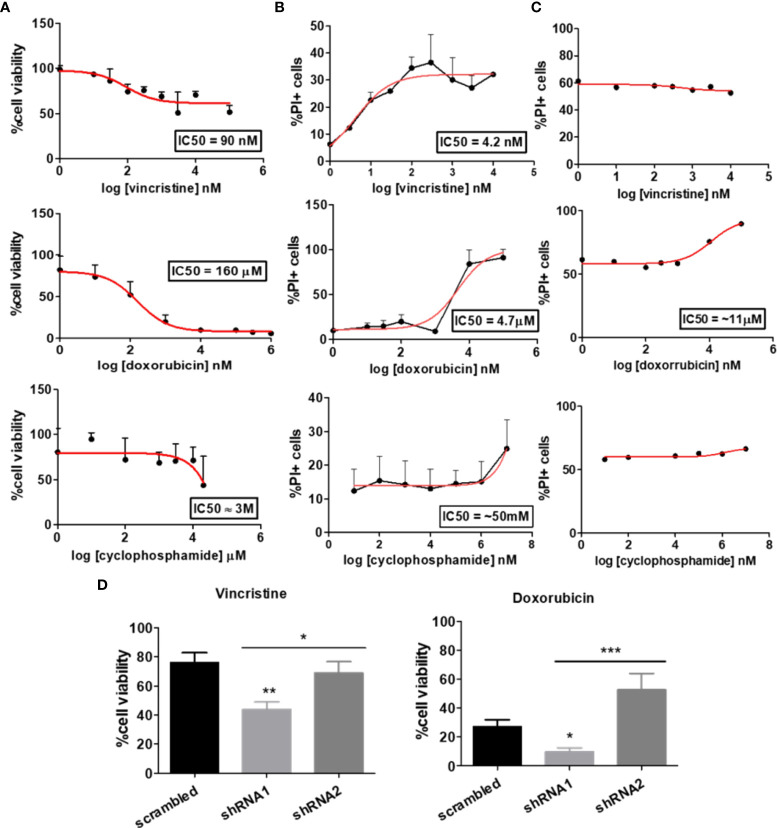
Dose‒response curve of chemotherapy in cells differentially expressing the P2X7 receptor and its isoforms. Dose–response curves of vincristine (n=6), doxorubicin (n=3) and cyclophosphamide (n=3) in neuroblastoma cells. **(A)** Viability assays and **(B)** cell death assays performed in ACN cells containing scrambled shRNA. **(C)** Drug-response curves of the chemotherapy drugs vincristine, doxorubicin and cyclophosphamide in HEK 293 cells obtained by PI staining quantification by flow cytometry. **(D)** Cell viability measurements of ACN cells expressing (scrambled), silenced for both A and isoforms (shRNA1) and silenced for B isoform (shRNA2) after treatment with vincristine or doxorubicin. Data normalized by the respective cell type untreated control (n>=3). *p<0.05; **p<0.01; ***p<0.001.

To investigate the influence of the P2X7 receptor and its isoforms on drug resistance and to confirm the function of each isoform, we chose two cell lines: a human neuroblastoma (NB) cell line (ACN cells) and HEK 293 cells. When submitted to 48 h treatments with drugs commonly used for NB treatment, ACN cells responded well to doxorubicin, i.e., presented a significant decrease in their viability upon treatment, as expected in chemotherapy, but not to cyclophosphamide – which is also expected as cyclophosphamide is a prodrug activated by hepatic metabolism -and had pronounced resistance to vincristine, reaching a plateau with a surviving ~50% population observed in both cell viability and cell death assays (**Figures 1A, B**). Similarly, HEK 293 cells did not show significant death rates upon vincristine or cyclophosphamide treatment (**Figure 1C**).

A shRNA-based silencing approach was used to selectively decrease the expression levels of P2X7 receptor isoforms. shRNA1, with a sequence designed to silence P2X7 receptor A and B isoforms, successfully decreased their expression levels, whereas shRNA2 selectively silenced B isoform, also according to what was expected based on its sequence (**Figure S1A**). HEK 293 cells, a cell type endogenously lacking P2X7 receptors (22), were transfected with the coding sequence of the isoform of interest, as described in the Methods section, generating three cell line subtypes: *mock* cells (control), HEK 293-A cells (P2X7A overexpression), and HEK 293-B cells (P2X7B overexpression) (**Figure S1B**).

In our experiments, shRNA1-silenced ACN cells were the most susceptible to both vincristine and doxorubicin treatments, presenting the highest decrease in cell viability (reminiscent viable population of 43.87% ± SE 5.2 (shRNA1) vs. 76.08% ± SE 6.75 (*scrambled*), and 69.08% ± SE 7.65 (shRNA2), *p*=0.0034) (**Figure 1D**). In contrast, shRNA2-silenced cells had the highest doxorubicin resistance (reminiscent viable population of 52.75 ± SE 11.37 (shRNA2) vs. 9.81 ± SE 2.76 (shRNA1), and 27.25 ± SE 4.72 (*scrambled*), *p*=0.0007), robustly demonstrated in several different culture conditions (**Figures 1D** and **S2A**).

For HEK 293 cells, isoform expression was also relevant to vincristine, but not to doxorubicin response. P2X7A overexpression was related to the highest decrease in cell viability upon vincristine treatment, reinforcing the previously demonstrated cell death-promoting roles of this isoform (**Figure S2B**).

Although these findings may sound controversial, they are indeed complementary. In the ACN NB-cell model, nonsilenced cells express the P2X7A isoform, but not exclusively: they also express P2X7B, and the trimeric receptor may occur in a heterogeneous composition, representing a scenario closer to reality. In the HEK 293 model, the isoforms occur alone and are overexpressed, isolating and highlighting the function of each isoform.

**Figure 2:**
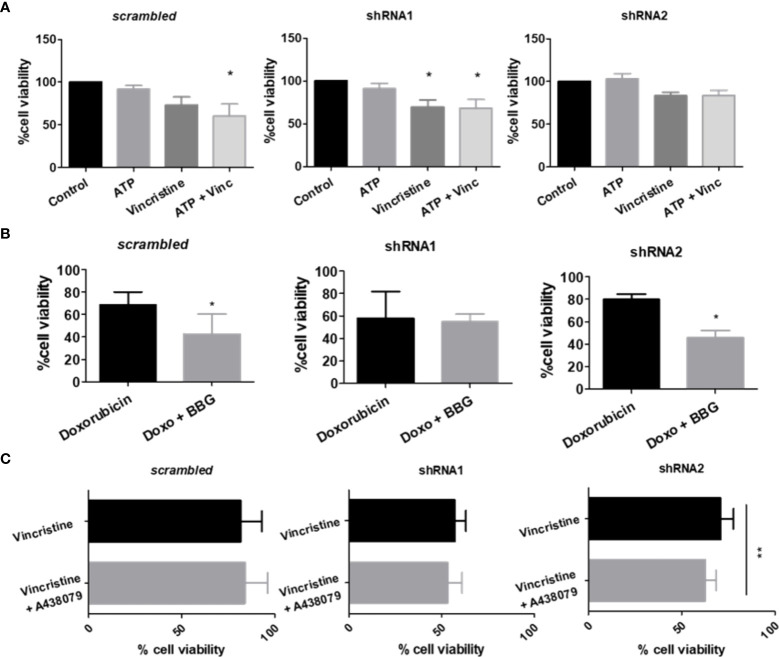
P2X7 receptor activity in response to chemotherapy. **(A)** Cell viability measurements of ACN cells stimulated or not with ATP, the endogenous P2X7 receptor agonist, alone or in combination with vincristine (n>=3). **(B)** Cell viability measurements of ACN cells stimulated with doxorubicin (doxo) in the presence or absence of the P2X7 receptor antagonist Brilliant Blue-G (BBG) (n>=4). **(C)** Cell viability measurements of ACN cells stimulated with vincristine in the presence or absence of the P2X7 receptor-specific antagonist A438079 (n>=4). Values were calculated as detailed in Methods section item 5. *p<0.05 and **p<0.01.

As P2X7 receptor activity in NB cells is less evident than that observed in the HEK 293 cell overexpression model, we treated ACN cells with P2X7 receptor agonists and antagonists. Vincristine treatment alone significantly decreased the viability of shRNA1 cells only (69.95% ± SE 7.99 vs. 100% in the control group, *p ≤* 0.05). However, the combination of vincristine and ATP, the endogenous P2X7 receptor agonist, promoted a similar effect in the nonsilenced cells (73.2% ± SE 9.46 vs. 60.73% ± SE 13.72 in the presence of ATP) (**Figure 2A**). This finding corroborates the role of the P2X7A isoform in promoting vincristine-induced death, as observed in HEK 293 cells overexpressing this isoform. However, ACN shRNA2 cells did not respond to vincristine either in presence or absence of ATP, suggesting that the P2X7B receptor alone is related to a drug-resistant phenotype (**Figure 2A**).

Increased doxorubicin resistance of nonsilenced or shRNA2-silenced cells was attenuated by antagonism with Brilliant Blue-G (BBG), while shRNA1-silenced cell drug sensitivity remained unchanged (**Figure 2B**). Regarding vincristine resistance, shRNA2-silenced cells showed decreased resistance upon selective antagonism with A438079, an effect that was not observed in nonsilenced cells, indicating that in this context drug resistance is related to the specifically assembled P2X7 receptor with the B isoform (**Figure 2C**).

### While the P2X7A isoform is critical for retinoid-induced neural differentiation of NB cells, the P2X7B variant is related to EMT marker upregulation in HEK 293 cells and enhanced EMT-induced drug resistance of NB cells

**Figure 3:**
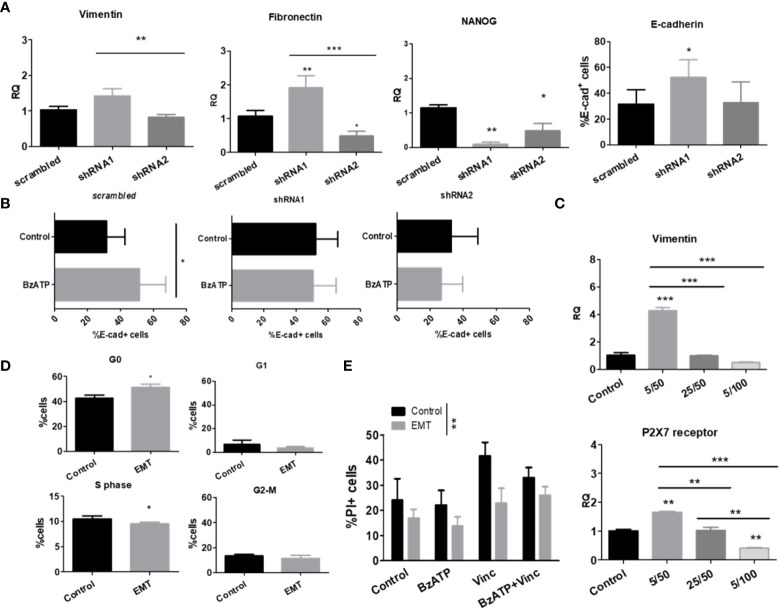
P2X7 receptor isoforms in epithelial-mesenchymal transition and stemness of neuroblastoma cells. **(A)** mRNA expression levels of vimentin, fibronectin and NANOG (n=3) and protein levels of E-cadherin (n=3) measured in scrambled, shRNA1 and shRNA2 cells. **(B)** E-cadherin protein levels measured by flow cytometry in BzATP-treated vs. nontreated ACN cells (n=3). **(C)** Vimentin and P2X7 receptor mRNA expression levels in scrambled ACN cells treated with combinations of low/high concentrations of TGF-β (5 or 25 ng/ml) and EGF (50 or 100 ng/ml) (n=3). **(D)** Cell cycle phase distribution of scrambled ACN cells treated with EMT inducers at optimal concentrations (TGF-β 5 ng/ml + EGF 50 ng/ml) (n=3). **(E)** Cell death measurements (PI-staining) of scrambled ACN cells treated with the P2X7 receptor agonist BzATP and/or vincristine, with or without treatment with EMT inducers at optimal concentrations (TGF-β 5 ng/ml + EGF 50 ng/ml) (n=3). *p<0.05; **p<0.01; ***p<0.001.

Because stemness and epithelial-mesenchymal transition (EMT) are closely related to chemoresistance (14), we characterized our cell lines, which differentially expressed P2X7 receptor isoforms, regarding the degree of differentiation and epithelial-mesenchymal phenotype transformation (**Figure 3**). We assessed the mRNA transcript levels of vimentin and fibronectin, which are mesenchymal phenotype markers, and E-cadherin protein levels, which are indicative of the epithelial-like phenotype (23). The mRNA levels of NANOG, a marker of neural progenitor cells (24), were used as an indicator of stemness for NB cells.

In ACN shRNA1-silenced cells, absence of the P2X7 receptor was related to increased expression of vimentin, fibronectin, and E-cadherin and decreased expression of NANOG (**Figure 3A**), pointing to a mixed EMT-MET (mesenchymal-epithelial transition) phenotype that cannot be restricted to a simple polarizing definition. The presence of P2X7B alone was related to decreased NANOG and fibronectin levels compared to nonsilenced cells, suggesting that these cells were not intrinsically more mesenchymal- or stem-like than those expressing both isoforms (**Figure 3A**).

Treatment of ACN cells with BzATP, a stable P2X7 receptor agonist, increased E-cadherin levels in control cells, an effect that was not observed in either shRNA1- or shRNA2-silenced cells, suggesting an epithelial-prone P2X7A-related effect (**Figure 3B**).

When treated with EGF and TGF-β, two known EMT-inducing factors, we observed that the lowest concentrations tested (50 ng/ml and 5 ng/ml, respectively) were optimal for inducing EMT in our model, producing the expected increase in vimentin expression levels. For this reason, we used these concentrations henceforth (**Figure 3C**).

This increase in vimentin expression upon treatment with EMT-inducing growth factors matched the enrichment of P2X7 receptor expression (**Figure 3C**) and increased cell quiescence (G0 state) in nonsilenced cells (**Figure 3D**), culminating in decreased cell death with vincristine treatment (**Figure 3E**).

Importantly, EMT made NB cells more resistant to death induced by treatment with vincristine alone or in combination with BzATP, the P2X7 agonist. This finding suggests that the mesenchymal phenotype overcomes P2X7A receptor activation, which produces death-promoting antitumor effects that were observed in NB cells expressing P2X7A and not treated with EMT-inducing growth factors (**Figure 2A**).

**Figure 4:**
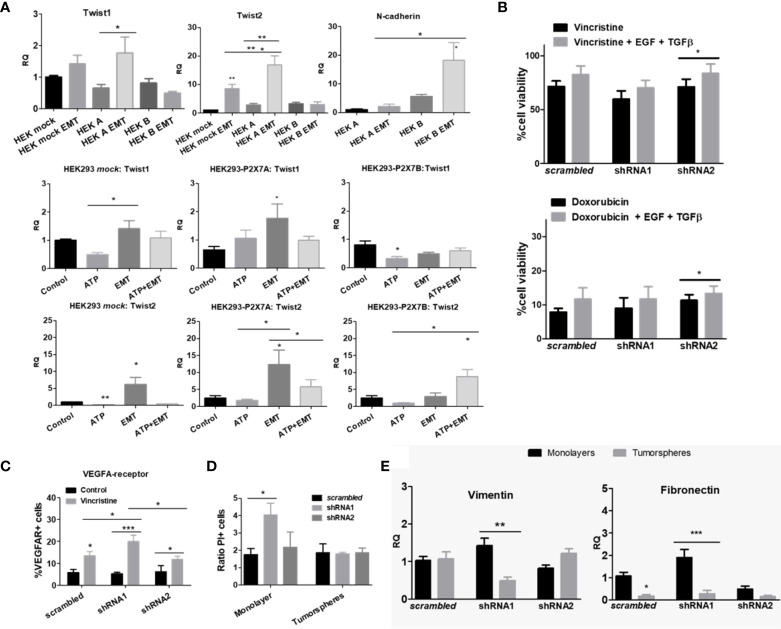
Epithelial-mesenchymal transition and stemness of cells cultured in monolayers or as tumorspheres. **(A)** mRNA expression levels of EMT-related transcription factors in HEK 293 cells, untreated, or treated with ATP and/or EMT inducers (EGF + TGF-β) (n=3). **(B)** Cell viability measurements of vincristine- (n>=5) or doxorubicin-treated (n=4) ACN cells in the presence or absence of EMT inducers. Values were calculated as detailed in Methods section item 5. **(C)** Expression levels of the VEGF-A receptor protein in ACN cells treated with or without vincristine. Measurements were performed by flow cytometry. (n=3) **(D)** Cell death ratio of ACN cells cultured in monolayers or as tumorspheres upon treatment with vincristine. For normalization of cell death intrinsic to tumorsphere manipulation, values were calculated as detailed in Methods section item 5. **(E)** mRNA expression levels of vimentin and fibronectin in ACN cells cultured in monolayers or as tumorspheres (n=3). *p<0.05; **p<0.01; ***p<0.001.

HEK 293-A cells shifted the expression levels of Twist-2, a transcription factor indicative of EMT. This effect was prevented by ATP treatment, which was also observed in *mock* cells, demonstrating that this ATP-induced effect was not related to the P2X7 receptor. When HEK 293-B cells were induced to undergo EMT, N-cadherin levels increased, but Twist2 levels increased only with concomitant addition of ATP, suggesting that P2X7B favors EMT and that direct stimulation with ATP enhances this effect, pushing cells toward a more mesenchymal phenotype. Because EMT did not occur in *mock* cells but only in P2X7B-overexpressing cells, we hypothesize that the acquisition of a mesenchymal phenotype is mediated by the B isoform, highlighting P2X7B as an EMT-favoring isoform (**Figure 4A**).

When ACN NB cells were EMT-induced and treated with either vincristine or doxorubicin, shRNA2 cells showed a significant increase in remaining cell viability (83.60% ± SE 8.79 vs. 70.95% ± SE 7.27 in nontreated cells, *p*=0.0443), an effect not observed in shRNA1 cells (**Figure 4B**), suggesting that cells predominantly expressing the P2X7B isoform undergo EMT more effectively and thus become more drug-resistant.

When the expression of VEGF-A receptor, an EMT marker, was assessed, we found no differences among the untreated cell types, but this receptor was enriched in all groups when submitted to vincristine treatment, reinforcing the relevance of EMT in vincristine resistance of NB cells (**Figure 4C**).

Cancer cells cultured as tumorspheres are often used as a model for cancer stem cell enrichment in culture. Therefore, we compared NB cells cultured in monolayers or as tumorspheres.

While shRNA1-silenced ACN cells cultured in monolayers responded better to vincristine when their counterparts were cultured as tumorspheres, isoform expression patterns were not relevant for drug responses, maintaining cell death rates within the same range for all cell types (**Figure 4D**). This means that whatever advantages P2X7-silenced cells had for treatment sensitivity, they were suppressed in tumorspheres, and these cells become as resistant as their nonsilenced or shRNA2-silenced counterparts. Therefore, phenotype modulation promoted by tumorsphere culture compensated for the absence of the P2X7 receptor. Thus, nonsilenced and shRNA2-silenced cells did not further increase drug resistance in face of these stimuli.

shRNA1-silenced cells, when cultured as tumorspheres, reduced vimentin expression levels to 33.8% of the levels observed in the monolayer culture (RQ of 1.42 ± SE 0.2 (monolayer) vs. 0.48 ± SE 0.09 (tumorspheres), *p*=0.0021) (**Figure 4E**), which may be interpreted as an indication of lower EMT grade. This may appear controversial to the fact that these cells respond poorly to vincristine in comparison to monolayer-cultured cells. In addition, both nonsilenced and shRNA1 cells had reduced fibronectin levels when cultured as tumorspheres (**Figure 4E**). However, in our experiments, epithelial phenotype did not seem to be a good predictor of drug response, and EMT induction was only effective in enhancing drug resistance in shRNA2 cells, linking EMT-induced drug resistance to the P2X7B isoform.

**Figure 5:**
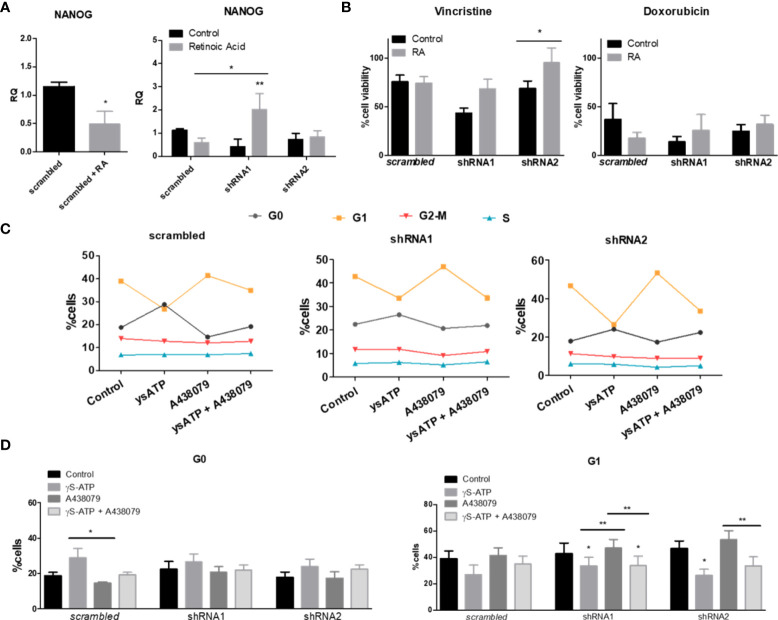
Retinoic acid-induced differentiation and cell cycle in NB cells differentially expressing P2X7 receptor isoforms. **(A)** NANOG mRNA expression levels in ACN cells treated with the neural differentiation inducer retinoic acid (RA) at a 5 µM concentration (n=4). **(B)** Cell viability measurements of ACN cells treated with RA and/or vincristine or doxorubicin (n=3). Values were calculated as detailed in Methods section item 5. **(C)** Complete representation of cell cycle phases of ACN cells after treatment with P2X7 receptor agonist and/or antagonist (n=5). **(D)** Distribution of ACN cells in cell cycle phases G0 and G1 after treatments with ysATP (an ATP analog resistant to hydrolysis), A438079 (selective P2X7 receptor antagonist) or both (n=5). *p<0.05; **p<0.01.

In order to investigate NB-cell differentiation ability, considering that the ACN cell line is poorly differentiated and mesenchymal-like per se (25), we stimulated cells with retinoic acid, a neural differentiation inducer (26). Retinoic acid treatment successfully decreased the expression levels of the neural progenitor marker NANOG in nonsilenced cells, as would be expected (RQ 1.42 ± SE 0.08 vs 0.48 ± SE 0.23 in retinoic acid-treated cells, *p*=0.0441). However, this reduction was not observed in shRNA1- and shRNA2-silenced cells; in fact, NANOG levels increased in shRNA1-silenced cells (RQ 0.41 ± SE 0.32 vs. 2.01 ± SE 0.69 in retinoic acid-treated cells, *p*<0.01) (**Figure 5A**). This finding suggests that the absence of the P2X7A isoform prevented neural differentiation of ACN cells, maintaining cells in an undifferentiated phenotype.

Retinoic acid treatment increased the vincristine resistance of cells lacking the P2X7A variant (**Figure 5B**), indicating that the absence of this isoform not only prevented neural differentiation but also enhanced resistance to vincristine, although not to doxorubicin (**Figure 5B**).

Cellular quiescence is a dormant-like cellular state that allows cells to evade cancer therapy, which largely targets proliferating cells (27). P2X7 receptor activation with γ-S-ATP, an ATP analog that is more resistant to hydrolysis, enriched quiescent populations of ACN cells and consequently decreased those in G1 phase (**Figures 5C, D**). However, the presence of the selective P2X7 receptor antagonist A438079 reversed this effect in cells expressing the P2X7A isoform, while in shRNA1- and shRNA2-silenced cells, the effect was persistent (**Figures 5C, D**), suggesting a P2X7A-related role.

### shRNA2-silenced cells exhibit decreased levels of autophagy markers, and its stimulation with rapamycin attenuates vincristine resistance, whereas starvation attenuates doxorubicin resistance

**Figure 6:**
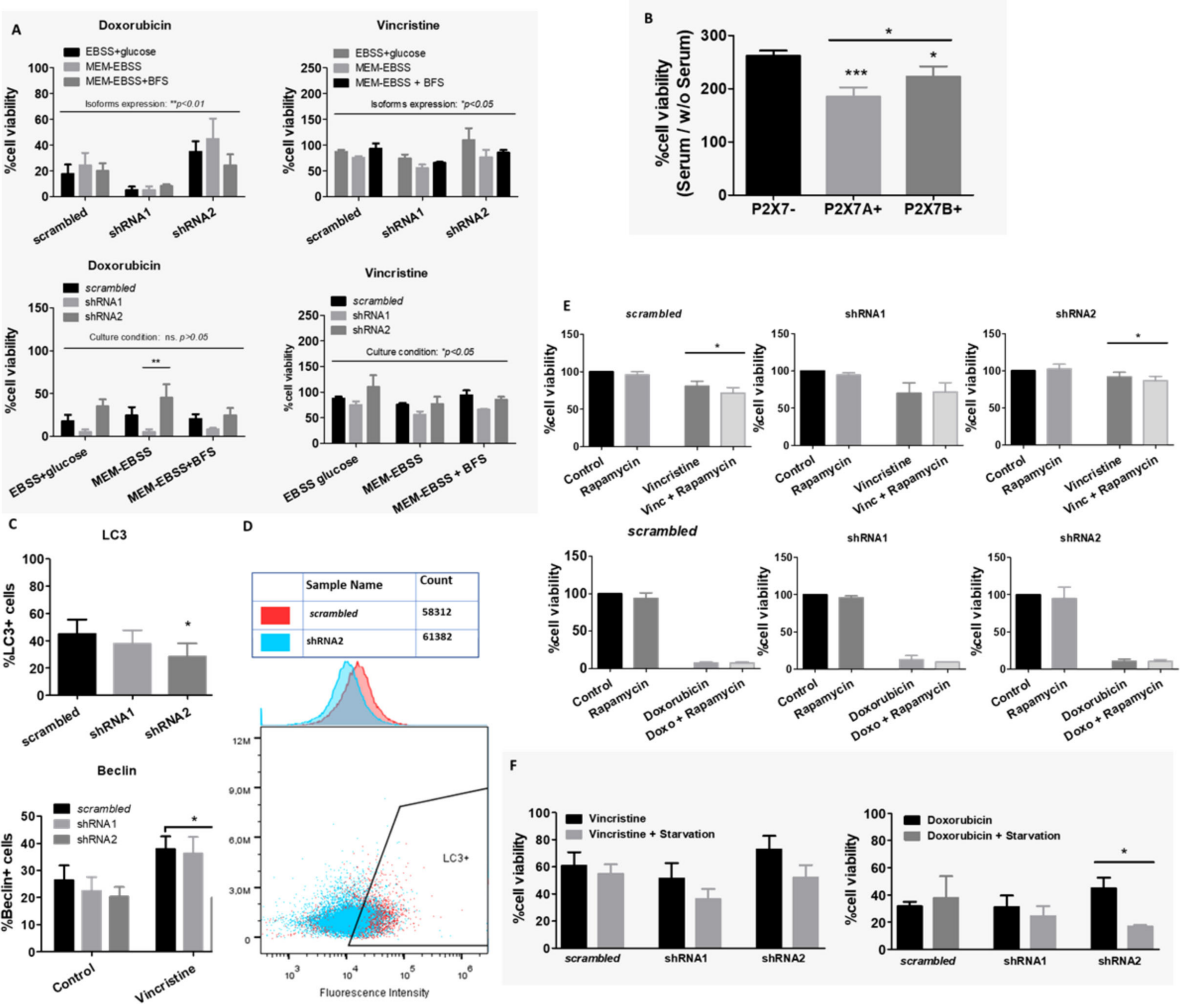
Culture conditions and autophagy in drug resistance. **(A)** Contributions of culture conditions and P2X7 receptor isoform silencing to the ACN cell drug response to doxorubicin (doxo) or vincristine (vinc) (n=3). **(B)** Cell viability percentage ratio of HEK 293 cells grown in serum-containing medium over serum deprivation, representing the serum-induced growth of HEK 293 cells according to the presence of P2X7 receptor isoforms (n=3). **(C)** Flow cytometry measurements of cell populations expressing the autophagy markers LC3 (n=4) and Beclin (n=3). **(D)** Representative cytometry dot plot of LC3+ cells in the scrambled or shRNA2 cell group. **(E)** Cell viability measurements of ACN cells treated with vincristine (n=5) or doxorubicin (n=3) in the presence or absence of rapamycin. **(F)** Cell viability measurements of ACN cells treated with vincristine or doxorubicin with or without serum starvation (n=3). Values were calculated as detailed in Methods section item 5. *p<0.05; **p<0.01; ***p<0.001.

When testing culture conditions, we observed that ACN NB cells appeared to respond well to doxorubicin under most culture conditions, whereas vincristine resistance was severely impaired. The expression patterns of isoforms in ACN cells appeared to determine both vincristine and doxorubicin responses (**Figure 6A**). Nonsilenced cells responded effectively to vincristine in EBSS-glucose and MEM-EBSS but not MEM-EBSS + FBS, pointing to a detrimental role of serum supplementation for drug efficacy when both P2X7 receptor isoforms are present, possibly indicating that growth factors and other molecules present in serum depend on the P2X7 receptor to induce proliferation or enhance cell survival. In shRNA1-silenced cells, drug response was better in MEM-EBSS and MEM-EBSS + FBS, reinforcing the hypothesis that the P2X7 receptor participates in the cellular response to serum compounds favoring drug resistance. Finally, shRNA2-silenced cells responded poorly under all conditions. For HEK 293 cells, culture conditions were the only determinant of doxorubicin effects (**Figure S2B**).

These differences suggest that metabolic pathways are essential for determining the drug resistance phenotype of both ACN and HEK 293 cells.

HEK 293 cells overexpressing P2X7A presented the lowest serum-induced growth ratio, followed by P2X7B-overexpressing cells, both of which were lower than *mock* cells ratio. This demonstrates that these cells can grow efficiently in the absence of serum, and as such, adding serum does not increase the growth efficiency as much (**Figure 6B**). The ability to grow in serum absence is a malignancy-related ability, as it allows cell survival in stressful situations. This is consistent with the increased drug resistance observed in P2X7 receptor-expressing NB cells, such as nonsilenced and shRNA2-silenced subtypes (**Figure 1E**).

Autophagy is tightly regulated and related to nutrient supply and stress stimuli. Therefore, we hypothesized that this important metabolic phenomenon might be involved in the observed drug resistance. To investigate this possibility, we measured autophagy markers in ACN cells and tested whether autophagy manipulation could modulate the drug response.

LC3 expression was decreased in shRNA2-silenced cells compared with the other cell groups, indicating low autophagic activity in cells that expressed isoform B only. In addition, Beclin-1 levels increased in control and shRNA1-silenced cells treated with vincristine, but this shift was not observed in shRNA2-silenced cells (**Figures 6C, D**). This finding suggests that shRNA2 cells do not exhibit increased autophagy in response to the stress stimuli induced by vincristine.

On the other hand, treatment with rapamycin, an autophagy inducer, decreased vincristine resistance in nonsilenced and shRNA2-silenced cells (**Figure 6E**). This finding indicates that compensation for the impaired autophagy observed in shRNA2 cells may be a strategy to attenuate drug resistance in this cell type. However, this was not the case for doxorubicin (**Figure 6E**).

When ACN cells were cultured under serum deprivation and in low glucose conditions, in comparison to high glucose plus serum supplementation, cell death rates in response to vincristine treatment increased independently of P2X7 receptor expression silencing (**Figure 6F**). However, for doxorubicin, this shift in cell death upon starvation was observed only in shRNA2-silenced cells (**Figure 6F**), suggesting that this attenuation in doxorubicin resistance may be related to starvation-induced autophagy upregulation.

### P2X7A has an efflux-preventing role, and P2X7B is associated with higher efflux activity, probably mediated by MRP-type ABC transporters

**Figure 7:**
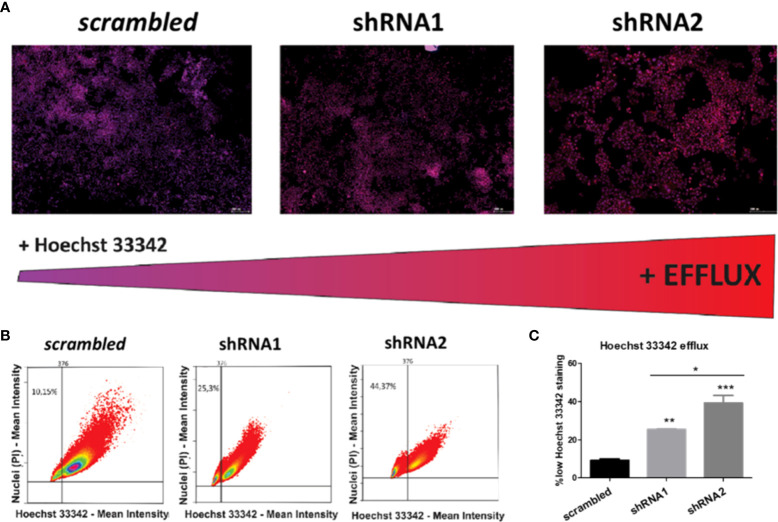
Effects of P2X7 receptor isoforms on the activity of drug efflux pumps in NB cells. **(A)** Fluorescence microscopy images of ACN cells differently silenced for P2X7 receptor isoforms costained with PI (nuclear marker, red) and Hoechst 33342 (efflux-prone dye, blue), obtained with a TissueFAXS^®^ fluorescence microscope. Scale bars = 200 µM. The redness of cell nuclei is indicative of lower Hoechst 33342 staining and thus higher efflux activity. **(B)** Dot plots quantifying the fluorescence intensity of PI and Hoechst 33342 on P2X7 receptor isoform-silenced or nonsilenced ACN cells. Thresholds set based on negative and positive control samples select a low Hoechst 33342-stained population. **(C)** Percentages of ACN cell populations with high efflux activity (low Hoechst 33342 staining). (n=3) *p<0.05; **p<0.01; ***p<0.001.

To investigate whether P2X7 receptor-mediated drug resistance is related to efflux pumps, an efflux activity assay was performed. shRNA2-silenced cells showed the highest efflux ability, followed by shRNA1-silenced cells (**Figure 7**). This suggests that P2X7A has an efflux-preventing role.

**Figure 8:**
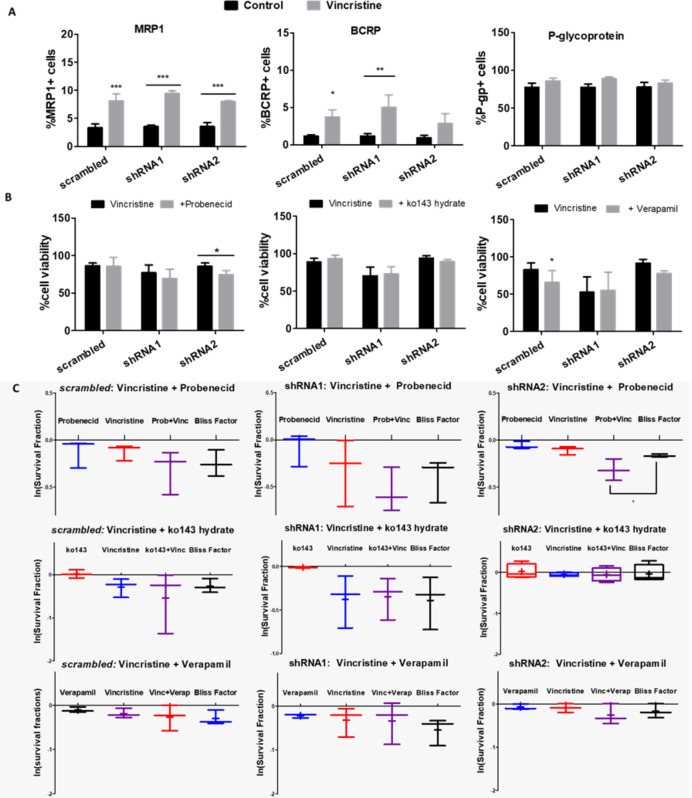
Functions of ABC transporters in NB cells differentially expressing A and B isoforms. **(A)** Flow cytometry measurements of MRP-1, BCRP and P-glycoprotein transporter-expressing ACN cells untreated vs. treated with vincristine (n>=3). **(B)** Cell viability values of ACN cells treated with vincristine and/or the ABC transporter modulator probenecid (MRP-1), ko143-hydrate (BCRP) or verapamil (P-glycoprotein) (n>=3). **(C)** Synergy investigation of vincristine combined with ABC transporter modulators in ACN cells using the Bliss Independence method (28), based on the natural logarithm of experimental survival fractions of populations treated with both drugs in combination, compared to the predicted survival fraction called Bliss Factor (n>=3). *p<0.05; **p<0.01; ***p<0.001.

Although P-gp is the most expressed efflux pump among those verified in this study, MRP pumps appear to be the most relevant for vincristine resistance in NB cells, as all ACN cell types exhibited increased MRP1 expression upon vincristine treatment (**Figure 8A**). In shRNA2-silenced cells, vincristine resistance was attenuated by MRP pump inhibition (probenecid) (**Figure 8B**), and Bliss independence analysis showed that probenecid acts synergistically with vincristine, reducing drug resistance (**Figure 8C**). P-gp inhibition only attenuated the drug resistance of shRNA1-silenced cells to doxorubicin, which does not seem to be a highly relevant resistance phenomenon because doxorubicin is highly effective in shRNA1-silenced cells (**Figure S3**). When the BCRP antagonist was administered concomitantly to anticancer drugs, no relevant differences were observed.

**Figure 9:**
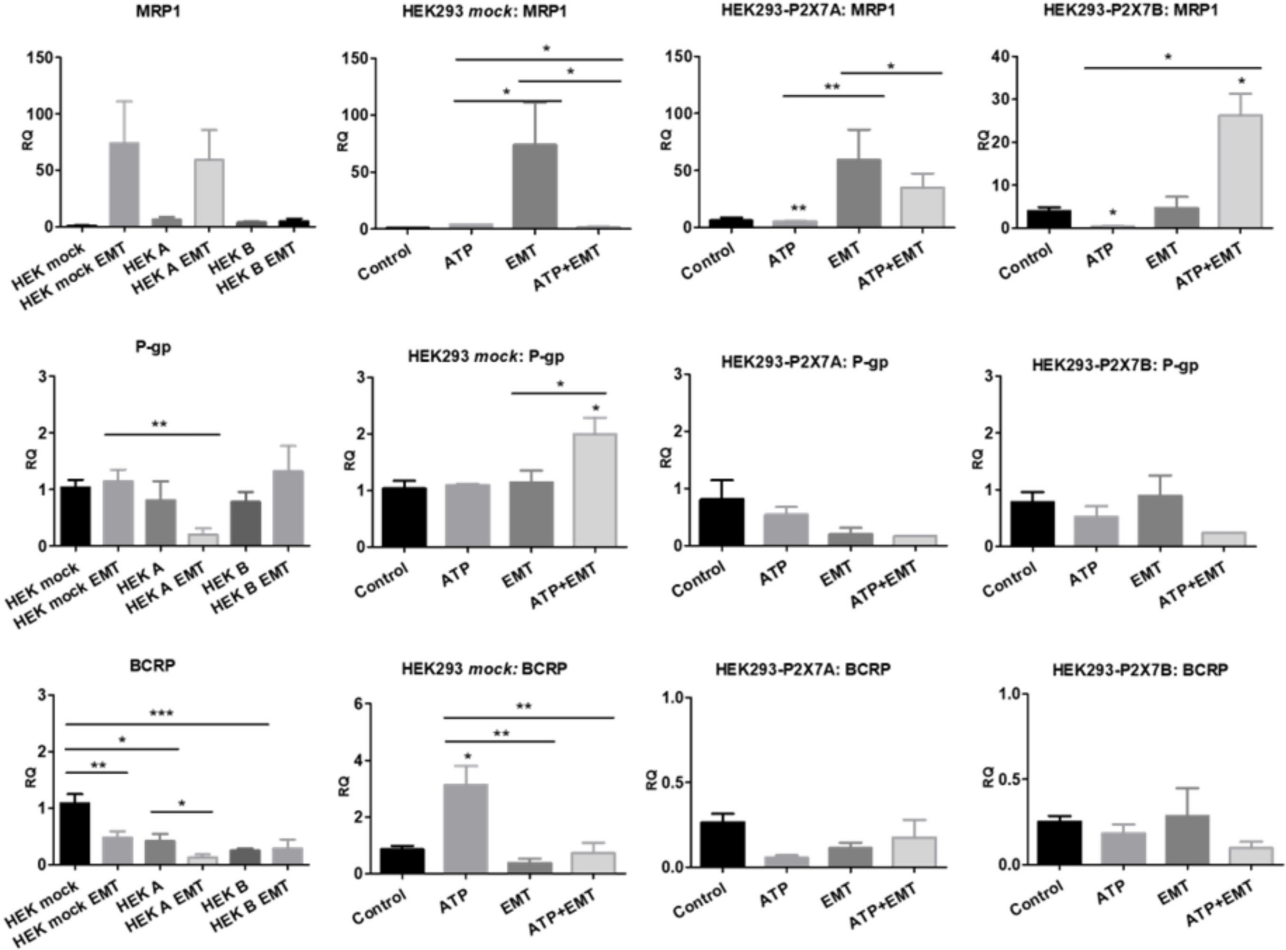
Contributions of P2X7 receptors and EMT to ABC transporter expression. mRNA expression levels of MRP1, P-glycoprotein (P-gp) and BCRP in HEK 293 cells treated or not treated with ATP and/or EMT inducers (EGF 50 ng/ml + TGF-β 5 ng/ml) (n>=3). *p<0.05; **p<0.01; ***p<0.001.

Upon EMT induction, HEK 293 *mock* cells raised MRP1 expression levels but decreased BCRP expression, and depended on ATP treatment to raise P-gp levels, an obviously not P2X7 receptor-mediated effect. When P2X7A was overexpressed, MRP1 and BCRP levels responded to EMT induction similarly to *mock* cells. However, P-gp levels were reduced, possibly due to P2X7A overexpression. In the case of P2X7B overexpression, the increase on MRP1 levels upon EMT induction depended on the presence of ATP (**Figure 9**), suggesting a critical role for the P2X7B variant in inducing MRP1 efflux pump expression, similar to the observed Twist2 expression patterns.

## Discussion

The therapeutic combination of vincristine, doxorubicin, and cyclophosphamide constitutes one of the cycles of induction therapy widely used for the treatment of high-risk neuroblastoma (29). These drugs have complementary mechanisms of action, and the cytotoxicity of the combination is based mainly on targeting DNA (30). When used individually, their effects are expected to diverge in terms of potency and specificity, considering the differences between their mechanisms and properties.

In our study, we observed high resistance of NB cells to vincristine, which is a tubulin-binding compound that acts through microtubule disruption (30). Vincristine is efficiently effluxed from cells *via* P-gp, leading to strong resistance to vincristine treatment in cell types overexpressing this efflux pump (30). Indeed, we observed that >90% of the ACN NB-cell population expressed P-gp, regardless of P2X7 receptor isoform expression patterns.

The responses observed upon agonism or antagonism of the P2X7 receptor are consistent with previous findings. While the P2X7 receptor is widely known as a cell death-promoting receptor, absence of the complete C-terminal tail prevents macropore opening, compromising the ability to induce cell lysis (14, 15, 31, 32). Whenever both isoforms A and B are expressed, they tend to coassemble, forming a heterotrimeric receptor. In this particular setting, the B isoform may enhance the responses mediated by P2X7A (16). Additionally, P2X7B expression alone is the phenotype mostly related to growth promotion (18), supporting our evidence implicating P2X7B isoform importance in drug resistance. Consistently, we observed that P2X7A-expressing HEK 293 cells have higher death ratios in response to chemotherapy drugs. ACN cells with basal expression of the P2X7 receptor were more drug resistant than their counterparts silenced for both A and B isoforms, also corroborating the survival and proliferation functions previously observed for endogenous expression of the P2X7 receptor (15, 33). Recent studies have suggested a role for P2X7B in resistance to daunorubicin and radiotherapy, such that cancer treatments that increase the extracellular ATP levels in the TME may lead to death of P2X7A-overexpressing cells while promoting the survival of cells overexpressing P2X7B (17, 34).

Here, we demonstrated that P2X7A is crucial for shifting the cell phenotype toward a more differentiated state, reducing cell stemness in response to retinoic acid. This finding is complementary to what was observed by Glaser *et al*: P2X7 receptor expression in embryonic cells was suppressed to allow neural differentiation (35). The most relevant isoform in this case is P2X7A, and when this isoform is absent in immature cells, differentiation is not triggered. Indeed, when used for neuroblastoma treatment, retinoic acid also faces resistance, a phenomenon termed retinoid resistance (36). If P2X7A expression relates to retinoid acid sensitivity, the detection of this isoform in the tumor mass might be a marker for guiding treatment decisions. Combining retinoic acid treatment with a P2X7 receptor agonist could also be a promising approach and is worth further investigation. Further preclinical studies could explore whether P2X7 receptor agonism together with retinoic therapy, prior to chemotherapy, would modulate the tumor mass toward a more drug-responsive phenotype, avoiding the selection of less differentiated resistant cells. Although there are still no specific agonists or antagonists for each isoform, both of them seem to be necessary for cell differentiation, meaning that a general agonist might work.

On the other hand, the P2X7B isoform was related to enhanced expression of EMT markers upon stimulation with well-known EMT inducers and increased EMT-triggered drug resistance. This finding is absolutely novel. Although relationships between EMT, invasiveness and the P2X7 receptor have been demonstrated (14, 37, 38), investigation of the involved isoforms has never been pursued. Because P2X7B is a main player in drug resistance and is also implicated in EMT, targeting this isoform in cancer seems promising for preventing EMT, thus overcoming drug resistance and tumor relapse.

shRNA2-silenced NB cells expressing only the P2X7B isoform showed decreased levels of autophagy markers. Induction of autophagy either pharmacologically or through starvation decreased resistance to the drugs used in our study, suggesting that this autophagy impairment may be an actual contributor to NB-cell resistance. Although it is usually thought that autophagy would be cytoprotective and thus enhance resistance, autophagy triggering is a mechanism of inducing cell death. Indeed, enhanced sensitivity to doxorubicin when autophagy is inhibited has been reported for several cancers (39) and increased autophagy levels in response to a class I phosphatidylinositol 3 kinase/mTORC1 inhibitor enhanced doxorubicin-induced apoptosis of NB cells (40), consistently to our observations. Regarding vincristine, inhibition of autophagy is also generally beneficial to decrease cancer cell drug resistance (30).

**Figure 10:**
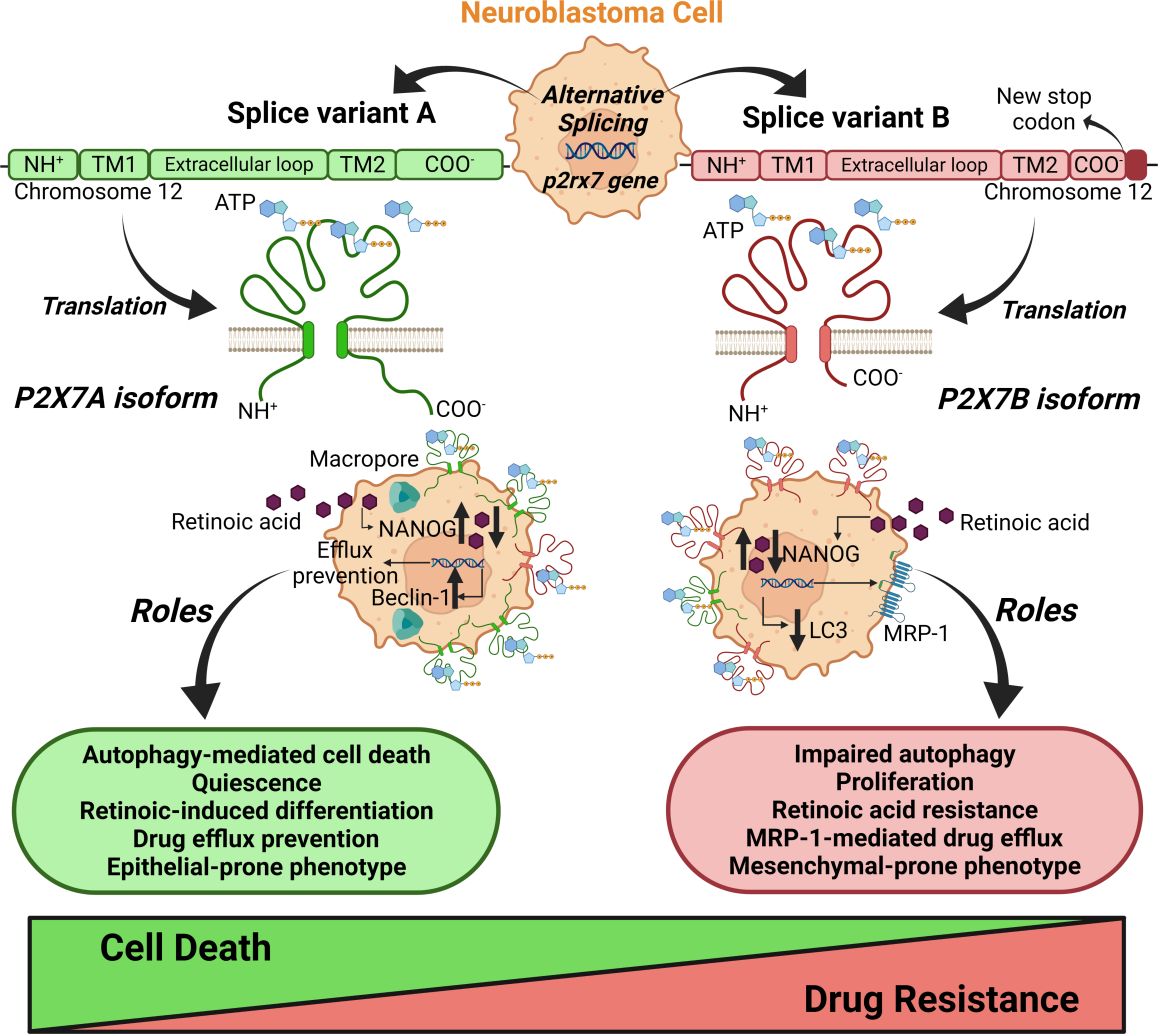
Schematic representation of the roles of splice variants of the p2rx7 gene in neuroblastoma chemoresistance. The alternative splicing of the p2rx7 gene localized on chromosome 12 generates several mRNA variants that are translated into nonfunctional or only two well-characterized functional ion channels, the A and B isoforms. P2X7A corresponds to the full-length variant, and P2X7B is produced by the retention of an intron containing a stop codon, which shortens the protein length. This truncated version lacks the C-terminal tail that has been described as crucial to macropore opening observed in cells expressing P2X7A. These structural differences in splice variants determine distinct contributions of the P2X7 receptor to neuroblastoma drug resistance. The P2X7A receptor is crucial for macropore-dependent autophagy-mediated cell death and quiescence induction and downregulates efflux pumps. P2X7A favors retinoid sensitivity for neuroblastoma cell differentiation and epithelial phenotype. The B isoform plays a primary role in cell proliferation (16) to favor drug resistance. The P2X7B receptor is important for resistance to retinoids, retaining cells in a stem-like phenotype, and efflux of drugs *via* MRP-type transporters. B isoform-expressing cells show impaired autophagy and a mesenchymal-prone phenotype. Taken together, these findings reveal counterbalancing roles for the A and B isoforms in neuroblastoma cells. Created with BioRender.com.

Previous evidence points to the P2X7 receptor either as a positive or negative regulator of autophagy (14, 41–45). In one study, P2X7 receptor agonism enhanced autophagic flux in a macropore-dependent way, leading to cell death, which was prevented by autophagy inhibition (42). In other words, the activation tonus of the receptor was decisive: while autophagy was increased upon tonic short-term stimulation with ATP, sustained stimulation with higher concentrations decreased autophagic flux (45). Once more, adding the isoforms to the equation helps clarify these divergences: while the P2X7A receptor is crucial to macropore-dependent autophagy-mediated cell death, as Fabbrizio *et al.* have shown, P2X7B receptor-expressing cells have lower autophagy levels and thus are less susceptible to autophagy-mediated cell death (**Figure 10**), as suggested by our data.

While P2X7A was important for preventing efflux, P2X7B related to the highest efflux phenotype in NB cells. This finding reinforces the perspective that for several functions, A and B isoforms have counterbalancing roles, as demonstrated for drug susceptibility, EMT, cell differentiation, and autophagy (**Figure 10**). This difference in efflux activity is most likely mediated by MRP-type pumps, as probenecid increased the susceptibility of P2X7B-expressing cells to vincristine. It has been demonstrated that treatment with retinoic acid decreases MRP1 expression levels in NB cells (46), which is consistent with our findings and helps conciliate all the evidence observed in the present work. If the absence of P2X7A maintains cells in a pluripotent state, as suggested by our data, and P2X7B expression increases drug resistance partly through MRP-type pump modulation, the inability of retinoic acid treatment to decrease pluripotency and thus MRP1 expression may be related to its inability to decrease drug resistance. In P2X7A variant-expressing cells, however, retinoic acid successfully decreased pluripotency and possibly MRP1 levels, as observed in the aforementioned study, explaining the reduced drug resistance.

## Conclusion

Tumor malignancy depends on a complex setting of characteristics shared by its constituent cells. Cancer cell stemness, mesenchymal-epithelial grade, efflux activity, metabolic regulation, and crosstalk with stromal cells are just a few countless processes that influence tumor aggressiveness. Following the conclusion that the P2X7 receptor isoform expression pattern was critical for drug resistance of NB cells, we observed some of the main candidates that could explain P2X7B-mediated drug resistance. Our results point to cooperatively built drug resistance, which is the result of many aspects that individually present only moderate effects but together build up a relevantly resistant phenotype. Given the pleiotropic role of the P2X7 receptor in modulating various cellular functions, this is not surprising. On the one hand, we demonstrated that P2X7A participates in triggering NB-cell differentiation, retinoid sensitivity and autophagy and downregulates efflux, and on the other hand, we showed the complementary role of P2X7B in suppressing autophagy, inducing drug efflux, and promoting EMT (**Figure 10**). The present work is thus highly relevant for resolving controversial findings in previous studies and proposing an integrated perspective to explore the P2X7 receptor.

## Data availability statement

The original contributions presented in the study are included in the article/**Supplementary Material**. Further inquiries can be directed to the corresponding author.

## Author contributions

VA-S: conceptualization; data curation; formal analysis; methodology; validation; visualization; roles/writing – original draft. CB: Data curation; Formal analysis; Methodology and figure conclusion; TG: Data curation; Formal analysis; Methodology; EA: Data curation; Methodology; Visualization; Writing – review and editing. HU: Conceptualization; Funding acquisition; Resources; Writing – review and editing. CL: Conceptualization; Data curation; Formal analysis; Funding acquisition; Investigation; Methodology; Project administration; Resources; Supervision; Validation; Visualization; Roles/Writing – original draft; Writing – review and editing. All authors contributed to the article and approved the submitted version.

## Funding

CL: São Paulo Research Foundation (FAPESP Project No. 2015/19128-2); HU: São Paulo Research Foundation (FAPESP Project No. 2018/07366-4).

## Acknowledgments

CL, TG, VA-S, CA, and HU thank the São Paulo Research Foundation and the National Council for Scientific and Technological Development (CNPq), Brazil for fellowships and financial support. CNPq project number 141264/2017-9.

## Conflict of interest

HU is a scientific adviser of TissueGnostics, Vienna, Austria, and receives consulting fees.

The remaining authors declare that the research was conducted in the absence of any commercial or financial relationships that could be construed as a potential conflict of interest.

## Publisher’s note

All claims expressed in this article are solely those of the authors and do not necessarily represent those of their affiliated organizations, or those of the publisher, the editors and the reviewers. Any product that may be evaluated in this article, or claim that may be made by its manufacturer, is not guaranteed or endorsed by the publisher.
