# Supplementary material for: P2X7 receptor isoform B is a key drug resistance mediator for neuroblastoma
Source: Front Oncol. 2022 Aug 25;12:966404. doi: 10.3389/fonc.2022.966404 (PMC9458077; doi:10.3389/fonc.2022.966404)
Supplement: Supplementary file 1 [file DataSheet_1.docx]

**SUPPLEMENTARY MATERIAL**

**
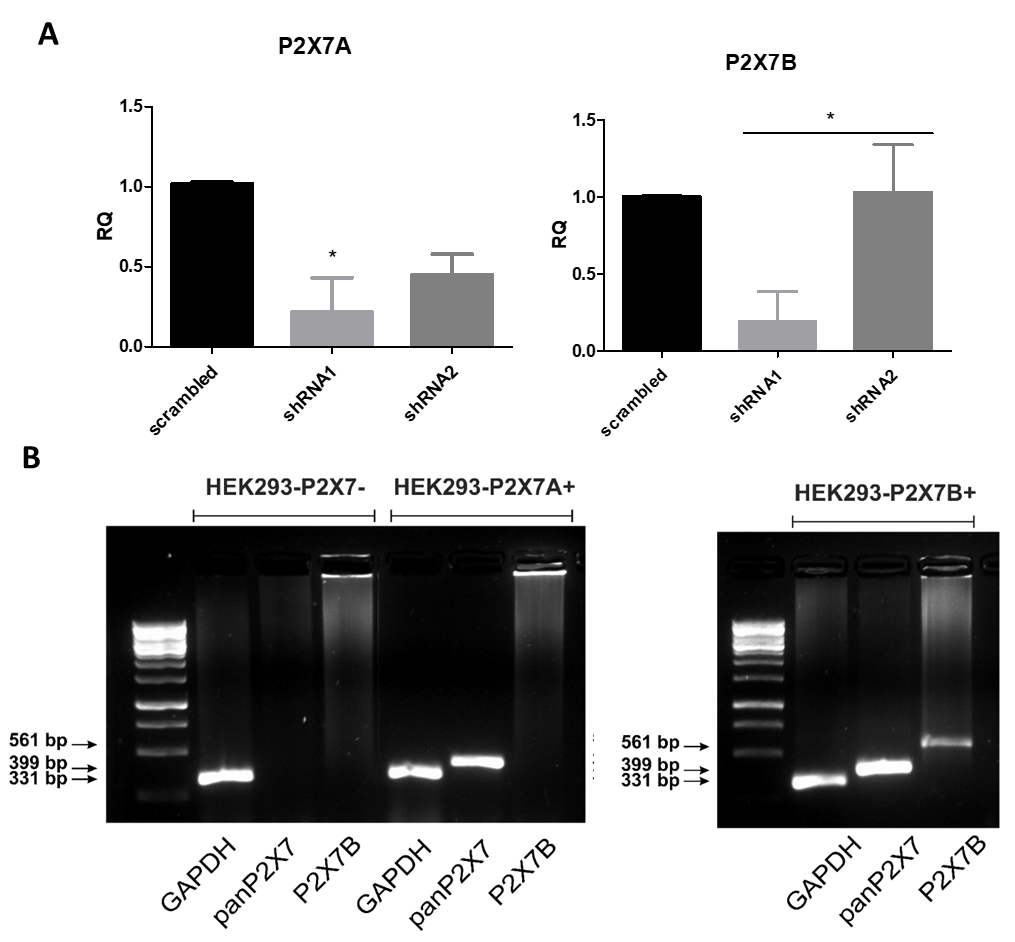
**

**Figure S1. Differential expression of isoforms A and B in neuroblastoma cells and HEK 293 cells**. A) mRNA expression levels of P2X7 receptor isoforms A and B in neuroblastoma cells stably transfected with the small hairpin RNAs scrambled, shRNA1 and shRNA2 (sequences described in Table 1); n=3. B) Agarose gel electrophoresis picture of PCR-reaction products performed for detection of P2X7 receptor isoforms in HEK 293 cells transfected for overexpression of isoforms A or B. *p<0.05 compare to scrambled.

**
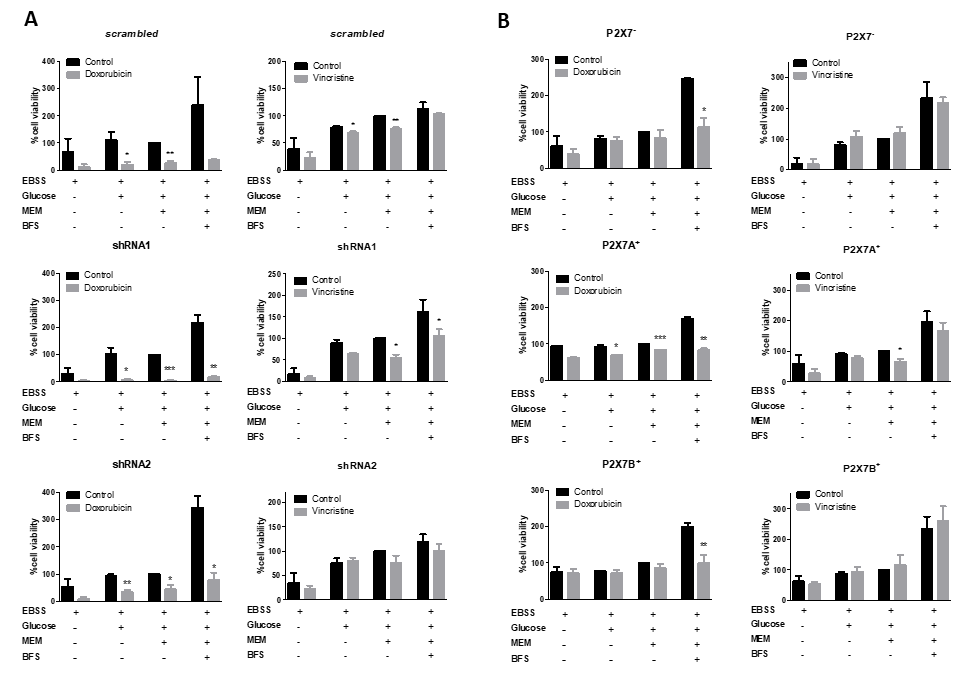
**

**Figure S2.** A) Cell viability values of ACN cells treated with vincristine or doxorubicin in progressively enriched culture conditions. MEM-EBSS medium was used as the control condition for normalization of cell viability values (n=3). B) Cell viability values of HEK293 cells treated with vincristine or doxorubicin in progressively enriched culture conditions. MEM-EBSS medium was used as the control condition for normalization of cell viability values (n=3). **p*<0.05; ***p*<0.01; ****p*<0.001.


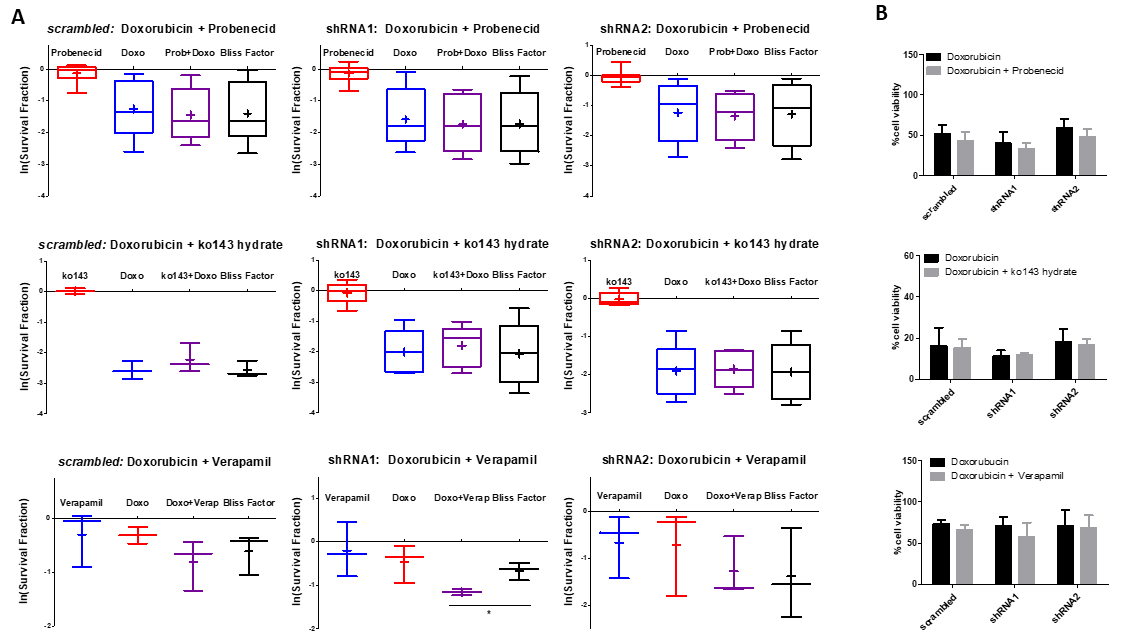


**Figure S3.** A) Synergy investigation of doxorubicin combined with ABC transporter modulators in ACN cells using the Bliss Independence method based on the natural logarithm of experimental survival fractions of populations treated with both drugs in combination compared to the predicted survival fraction called the Bliss Factor (n>=3). B) Cell viability values of ACN cells treated with vincristine and/or the ABC transporter modulators probenecid, ko143-hydrate or verapamil (n>=3). **p*<0.05; ***p*<0.01; ****p*<0.001.
